# Supplementary material for: A CD47-associated super-enhancer links pro-inflammatory signalling to CD47 upregulation in breast cancer
Source: Nat Commun. 2017 Apr 5;8:14802. doi: 10.1038/ncomms14802 (PMC5382276; doi:10.1038/ncomms14802)
Supplement: Supplementary Information — Supplementary Figures and Supplementary Tables [file ncomms14802-s1.pdf]

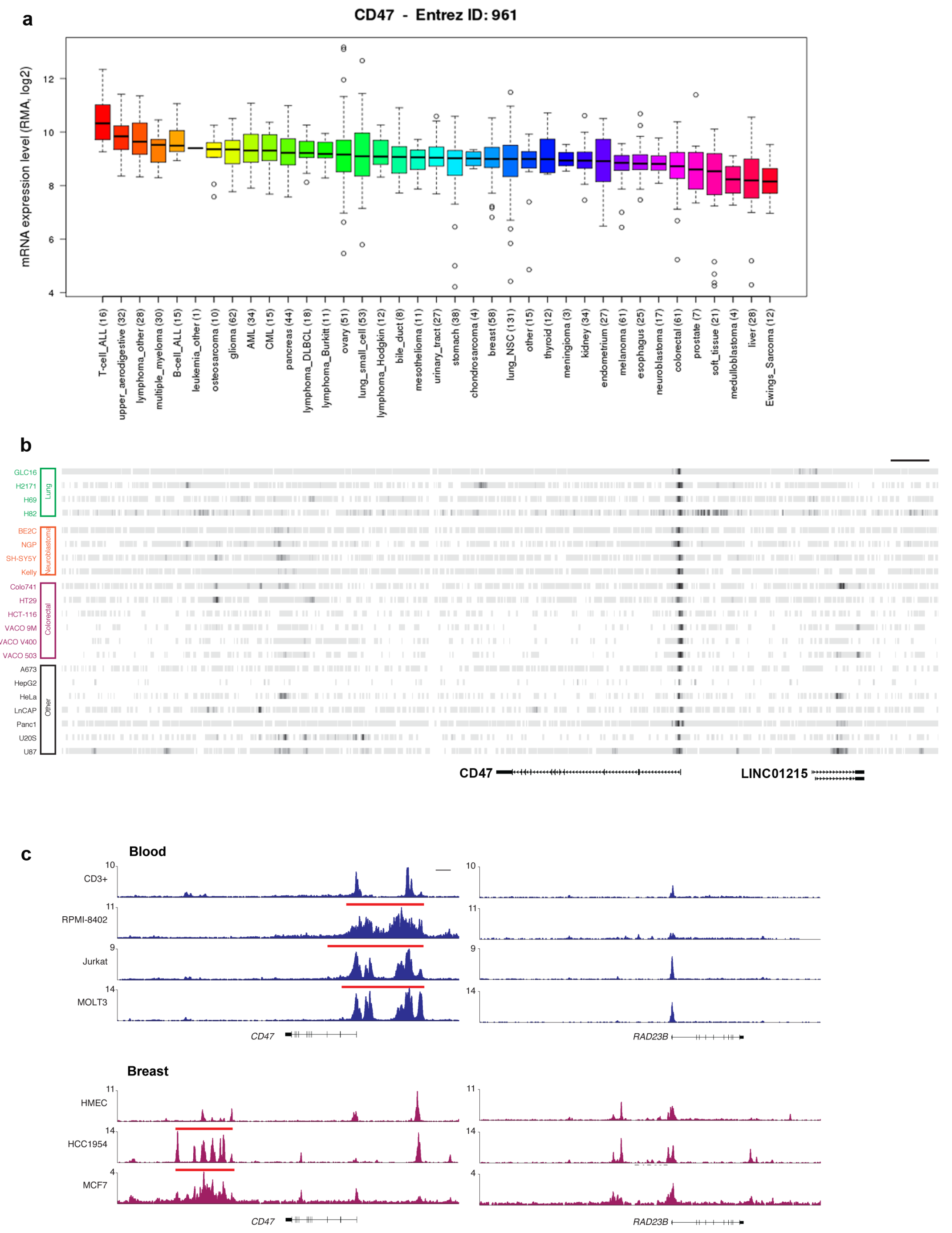

Supplementary Figure 1. Cancer cell lines RPMI-8402, Jurkat, MOLT3, LY4, HCC1954 and MCF7 expressing high *CD47* levels have *CD47* associated super-enhancers (a) *CD47* expression profile across different cancer cell types based on publicly available microarray data. Left to right: cancer cell types that express the highest to lowest *CD47*. Cancer lines having SEs associated with

CD47, such as Jurkat (T-ALL) and MCF7 (breast cancer) are within the cancer types that express higher levels of *CD47*, while VACO400, VACO503, and VACO9M (colorectal) and HepG2 (liver) lines are within the cancer types, which express *CD47* at lower levels. (b) Heat map representing the distribution of H3K27ac enriched (grey to dark grey) regions within the human *CD47* genomic locus show different cancer cell lines lacking *CD47* SEs. (c) H3K27ac binding profiles shows that a SE (red line on top) is associated with *CD47* in T-ALL cancer cell lines RPMI-8402, Jurkat and MOLT3, but not in CD3+ T (normal) cells. Analyses of H3K27ac across different breast cancer types show that cell lines HCC1954 and MCF7 have a SE (red line on top) associated with *CD47* when compared to a non-tumorigenic human mammary epithelial cell line (HMEC). H3K27ac binding profiles show typical enhancers at the RAD23B gene for size comparison between SEs and typical enhancers (right panel). Scale bar: 10kb.

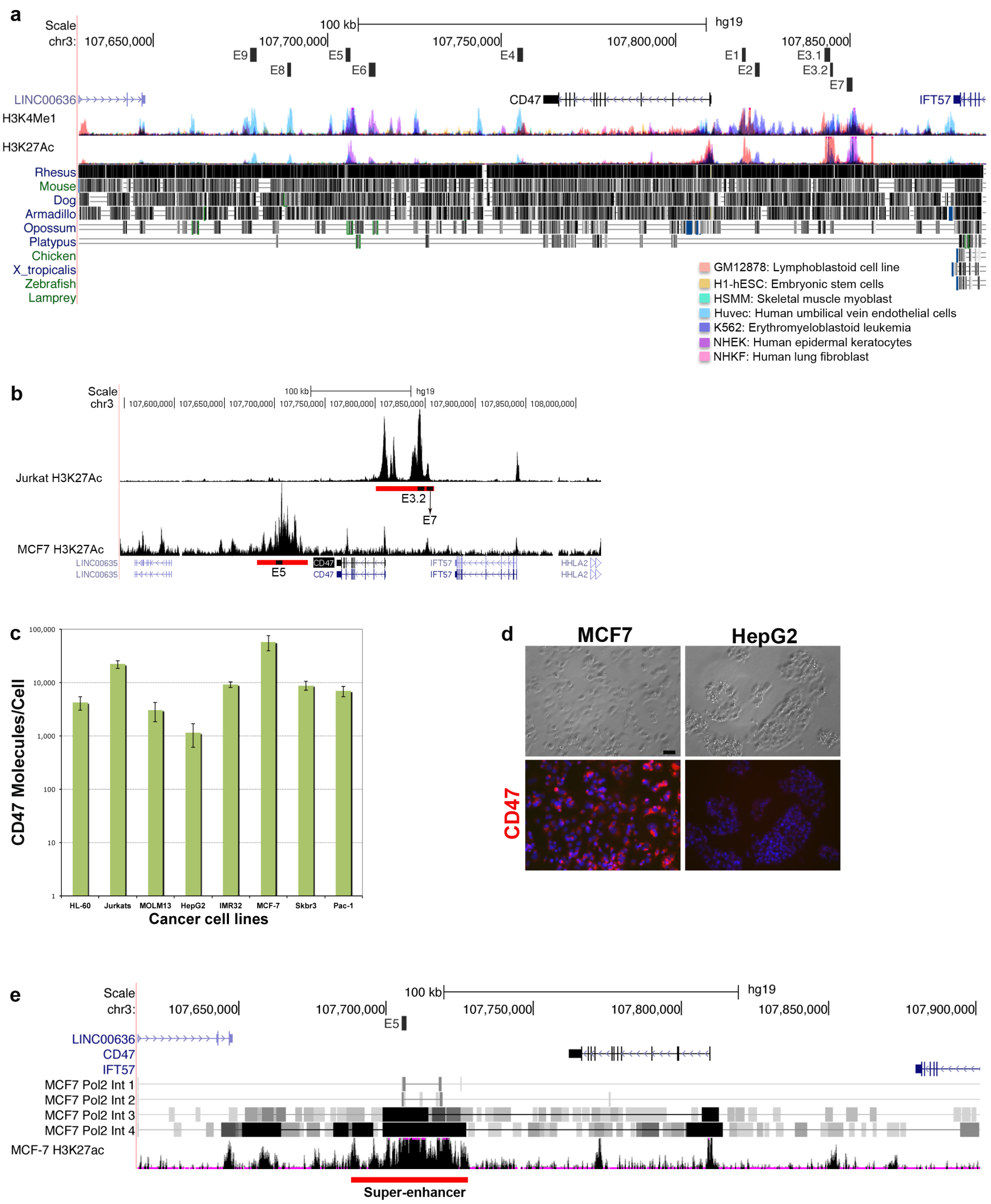

Supplementary Figure 2. Locating *CD47* active constituent enhancers in MCF7 breast cancer and Jurkat T-ALL cells (a) Location of putative *CD47* active enhancers across *CD47* genomic locus. Active enhancers were predicted based on: i) H3K4Me1 and H3K27ac ChIP-Seq data publicly available for 7 different cell lines (colored peaks); and ii) genomic conservation across different species (black bars). Thus, 9 regions were predicted (E1-E9, black blocks). (b)

CD47 SE E3.2 and E7 constituents are located within an upstream SE in the Jurkat (T-ALL) cell line; and the *CD47* SE E5 constituent is located downstream in MCF7 cells. Bottom red lines mark super-enhancers while the black blocks within show the location of functional constituent enhancers. (c) Quantification of CD47 protein levels in cancer cell lines, by measuring CD47-PE antibody bound per cell (ABC), shows that CD47 protein levels are higher in Jurkat (T cell leukemia), IMR32 (neuroblastoma) and **MCF7** (breast cancer) cell lines when compared to others cancer cell lines. Cancer cell lines analyzed: HL-60, Jurkat and MOLM13: leukemia. HepG2: hepatoma. IMR32: neuroblastoma. MCF7 and SKBR3: breast cancer and Panc1: pancreatic carcinoma. Protein amounts were measured by using a fluorescent Phycoerythrin (PE) conjugated antibody against CD47 and flow cytometry. (d) Immunohistochemistry confirms that protein levels of CD47 are high in MCF7 and low in the HepG2 cell line. Scale bar: 100um. (e) ChIA PET publicly available data shows interaction between the downstream SE constituent E5 and *CD47* promoter in MCF7 cells (tracks Int1 and Int2, grey blocks; tracks Int3 and Int4, black blocks). Black peaks show H3K27ac enriched regions. Gene names for figures a, b and d are in black or blue.

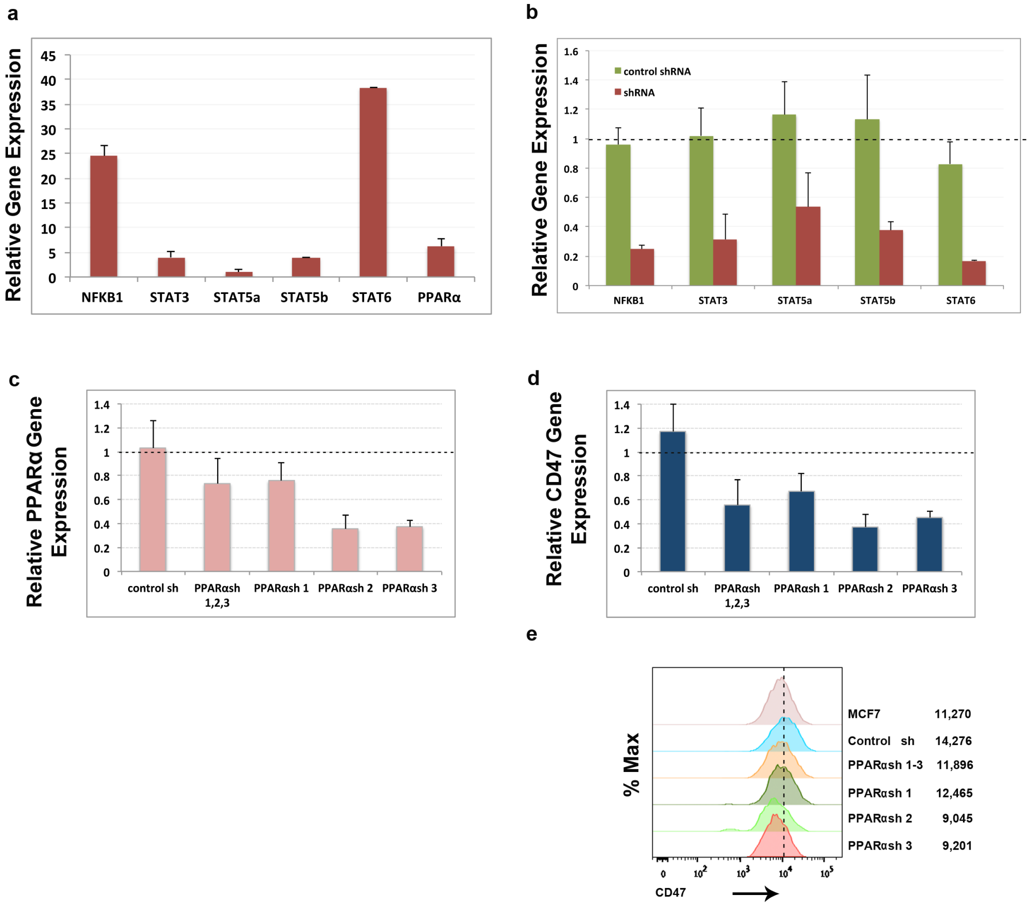

Supplementary Figure 3. Expression and knock-down efficiency profiles of the CD47 candidate transcriptional regulators NFKB1, STAT3, STAT5(a & b), STAT6 and PPARα (a) qPCR gene expression profile of *CD47* E5 binding candidate transcription factors confirm that *NFKB1, STAT3, STAT5*(*a* & *b* variants*)*, *STAT6*, and *PPARα* are present in MCF7 cells at different levels. (b) shRNA based knock-down efficiently reduces the transcript expression of the target candidate genes *NFkB1, STAT3, STAT5*(*a* & *b* variants). (c) PPARα shRNA hairpins 2 and 3 efficiently knock-down the *PPARα* transcription factor. On the contrary, PPARα shRNA hairpin 1 or the combination of the 3 hairpins are not as effective. (d) A greater reduction on *CD47* expression is achieved when knocking-down *PPARα* by using shRNA hairpin 2 or 3 when compared to hairpin 1 or the combination of 3 hairpins. (e) Similarly, a greater reduction on CD47 protein levels is observed after knocking-down *PPARα* by using shRNA hairpin 2 or 3.

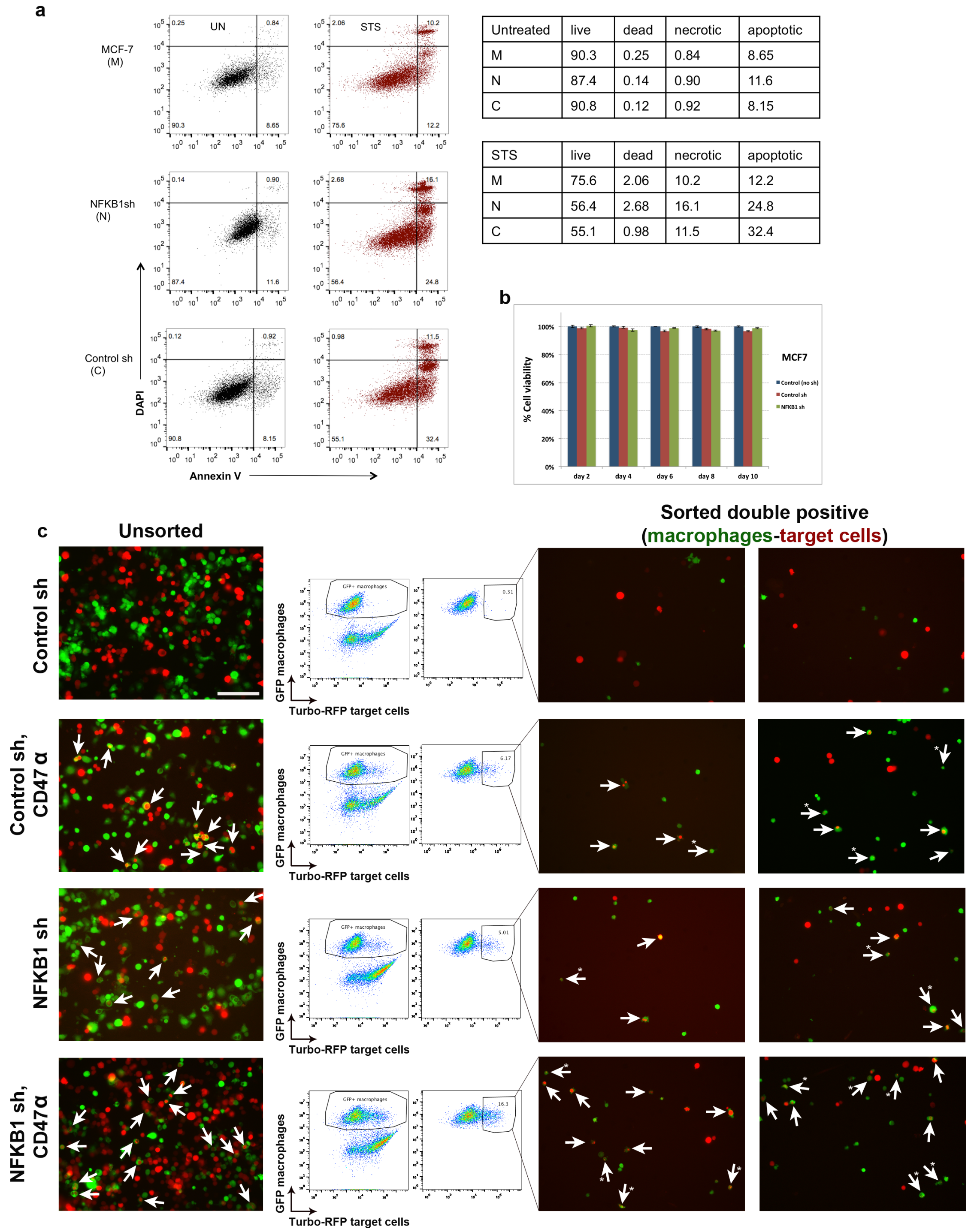

Untreated	live	dead	necrotic	apoptotic
M	90.3	0.25	0.84	8.65
N	87.4	0.14	0.90	11.6
C	90.8	0.12	0.92	8.15

STS	live	dead	necrotic	apoptotic
M	75.6	2.06	10.2	12.2
N	56.4	2.68	16.1	24.8
C	55.1	0.98	11.5	32.4

Supplementary Figure 4. Knocking-down NFKB1 by shRNAs does not affect viability and increases phagocytosis of MCF7 cells (a) MCF7 cells (M) untreated (UN), treated with NFKB1 shRNA (N) or control shRNA (C) were stained with Annexin-V (conjugated to AF-488) antibody to measure early

apoptosis and DAPI (4',6-diamidino-2-phenylindole) to discriminate dead cells. No significant percentage increase in dead (DAPI+, Annexin V-), necrosis (DAPI+, Annexin V+) or apoptosis (DAPI-, Annexin V+) was observed in shRNAs treated MCF7 cells when compared to the untreated and to staurosporine (STS) treated MCF7 cells. (b) Percentage cell viability of untreated, control shRNA or NFKB1 shRNA treated MCF7 cells calculated by dividing the total number of live cells by the total number of cells multiplied by 100. Number of live cells was determined after staining with trypan blue and counting unstained cells for 5 passages. No significant difference was observed among the NFKB1 shRNA, control shRNA treated and untreated MCF7 cells. (c) Representative images of a phagocytosis assay FACS analysis confirming that GFP macrophages (in green) engulf MCF7 target cells (in red) after treating target cells with CD47 blocking antibody or turboRFP-NFKB1 shRNA or both. Panels to the left of the FACS plots, show incubated macrophages with target cells (without or with treatment) prior FACS analyses and sorting. Panels to the right of the FACS plots show sorted double positive red and green cells (without or with treatment) approx. two hours after sorting. White arrows point at green macrophages that are engulfing or have engulfed red MCF7 target cells. White arrows with asterisks point at examples of target cells being digested by macrophages based on a decrease of turboRFP signal. Scale bar: 100um.

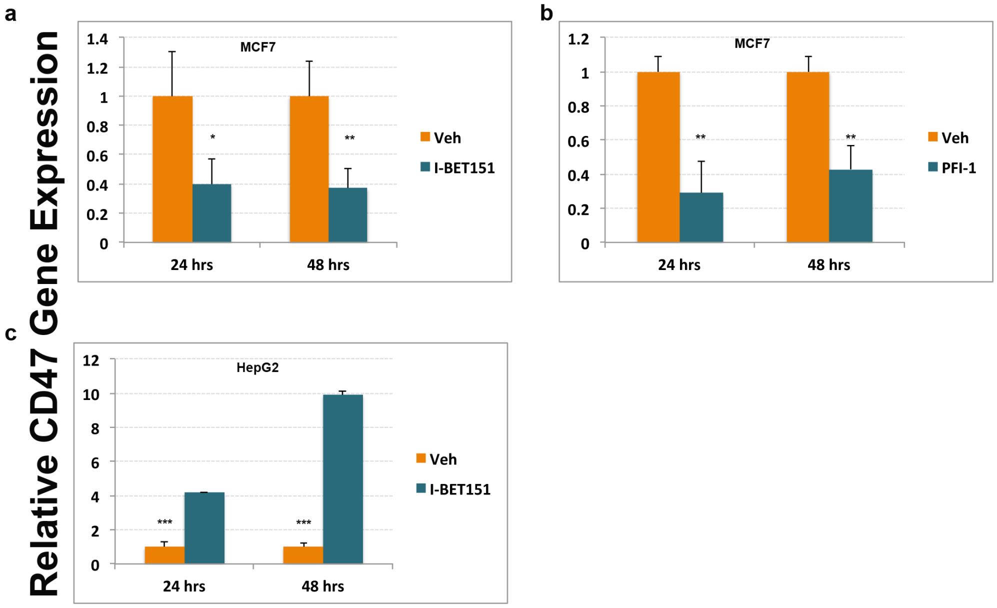

Supplementary Figure 5. BRD4 inhibition reduces *CD47* expression (a,b) Treating MCF7 cells with the BRD4 inhibitors I-BET151 (100uM) and PFI-1 (500uM) reduces *CD47* gene expression. N=3 samples. (c) On the other hand, treating the negative control, HepG2 cells (which lack SEs) with I-BET151 (100uM) inhibitor increases *CD47* gene expression. N=3 samples. Values represent mean + s.d. Student's unpaired t test for independent samples was performed. ***P <0.01, **P<0.05, *P<0.1.

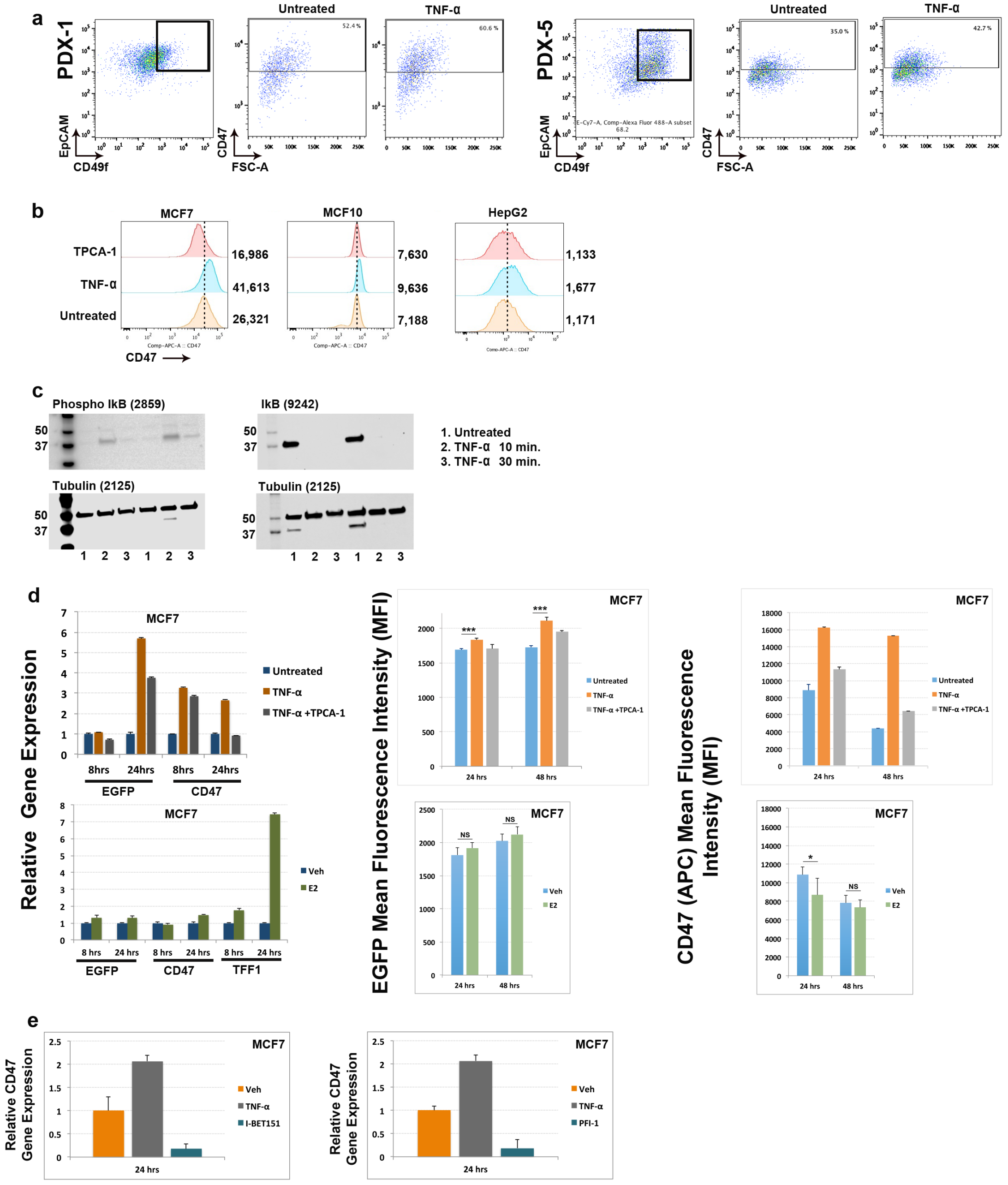

Supplementary Figure 6. TNF pathway stimulation increases CD47 levels through the activation of *CD47* E5 constituent enhancer in breast cancer (a) Representative FACS dot plots of 2 patient-derived tumor xenograft (PDX) samples show that CD47 protein levels increase in the tumorigenic population ($EpCAM^{Hi}$, $CD49f^{Hi}$) 24 hours after TNF-α stimulation. (b) Inhibitor TPCA-1 prevents TNF-α induced CD47 upregulation by blocking the NFKB1 activator, IKK2, in MCF7, MCF10 and HepG2 cancer cell lines. Mean values are shown to

the right of each histogram. (c) Western blot analyses demonstrate that the NFKB1 inhibitor, IkB, is phosphorylated (left panel) and degraded (right panel) after stimulating MCF7 cells with the TNF-α ligand. (d) Treating MCF7 cells carrying the *CD47* E5-TK-EGFP construct with the TNF-α ligand increases EGFP transcript and protein expression and such effect is abolished by the inhibitor TPCA-1, thus resembling endogenous CD47 response to TNF-α stimulation and IKK2 inhibition by TPCA-1 (upper graphs). The lower graphs show that while treatment of *CD47* E5-TK-EGFP MCF7 cells with β-Estradiol (E2) increases endogenous gene expression of the control Trefoil Factor (*TFF1*), a transcription factor that responds to Oestrogen Receptors activation, the E2 treatment does not affect EGFP reporter or endogenous CD47 (transcript and protein expression). N=3 samples. Values represent mean + s.d. Student's unpaired t test for independent samples was performed. ***P <0.01, *P<0.1. N.S = not significant. (e) TNF-α mediated *CD47* upregulation in MCF7 cells is inhibited upon treatment with I-BET151 or PFI-1 (bromodomain inhibitors).

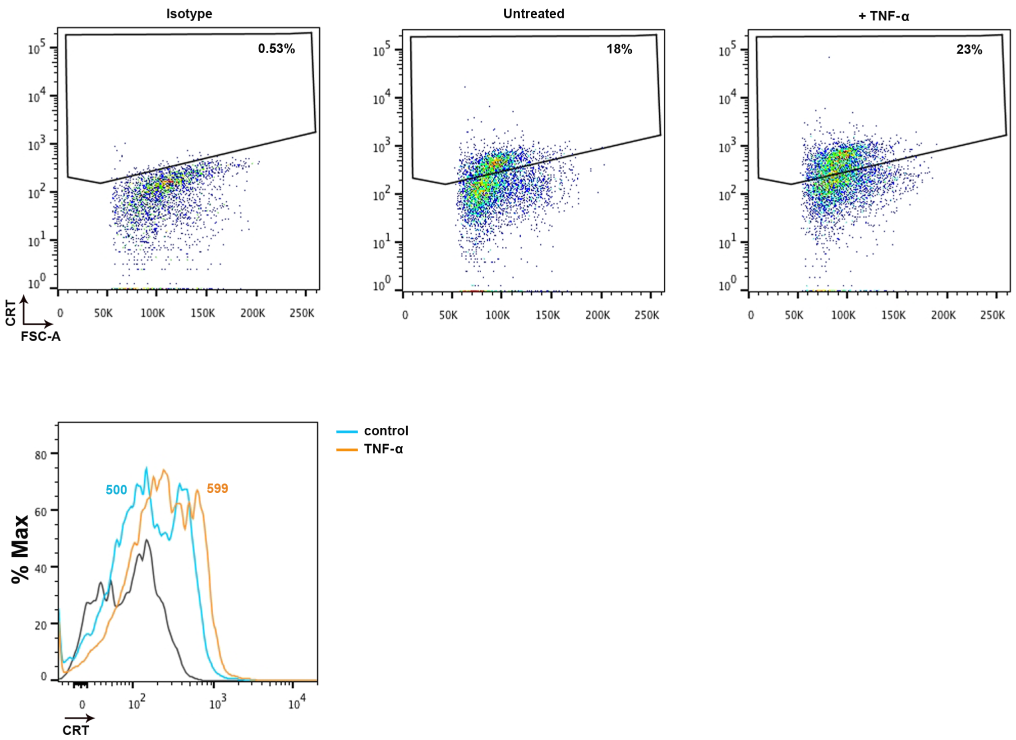

Supplementary Figure 7. Calreticulin expression increases in MCF7 cells upon TNF-α treatment Calreticulin, a prophagocytic cell surface molecule is expressed by MCF7 cells and is slightly increased after the cells are stimulated with TNF-α (orange histogram) for 48hrs. Grey histogram represents the isotype control.

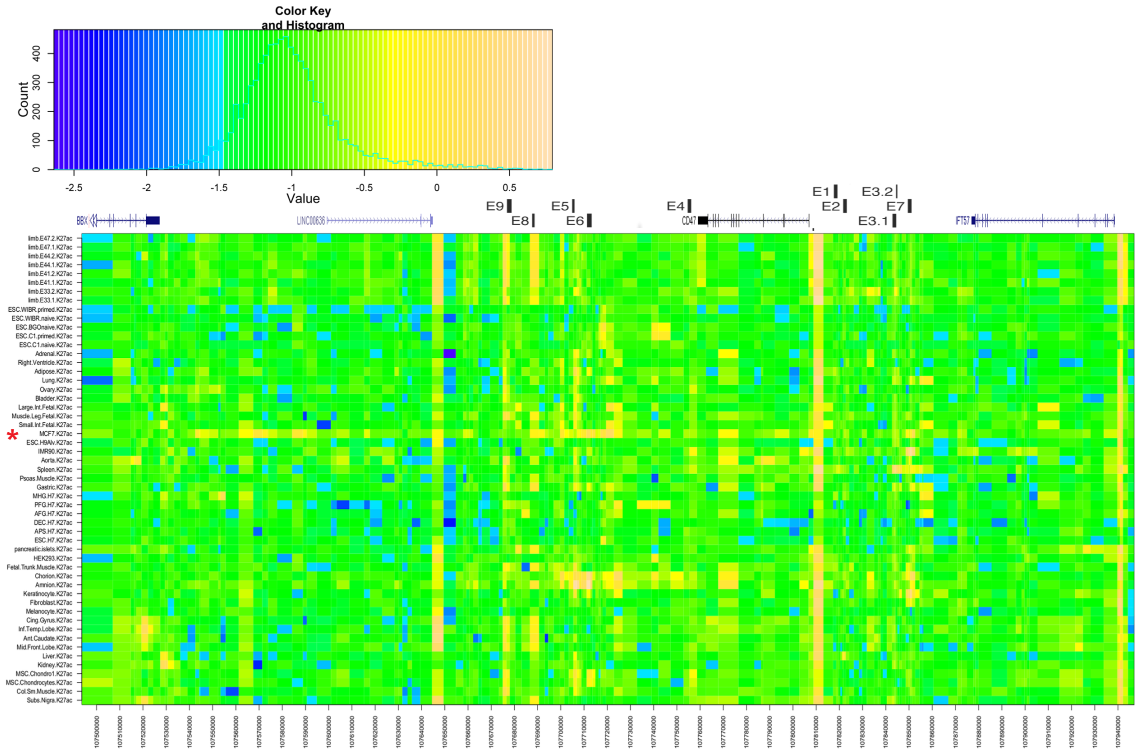

Supplementary Figure 8. H3K27ac global analysis covering the *CD47* locus in different tissue and cell types Heat map showing the distribution of H3K27ac enriched (yellow to orange) regions within the human *CD47* genomic locus in different tissue and cell types compared to the H3K27ac clustering occurring in MCF7 cells (red asterisk). This global analysis was performed using publicly available ENCODE and limb data from the Noonan laboratory.

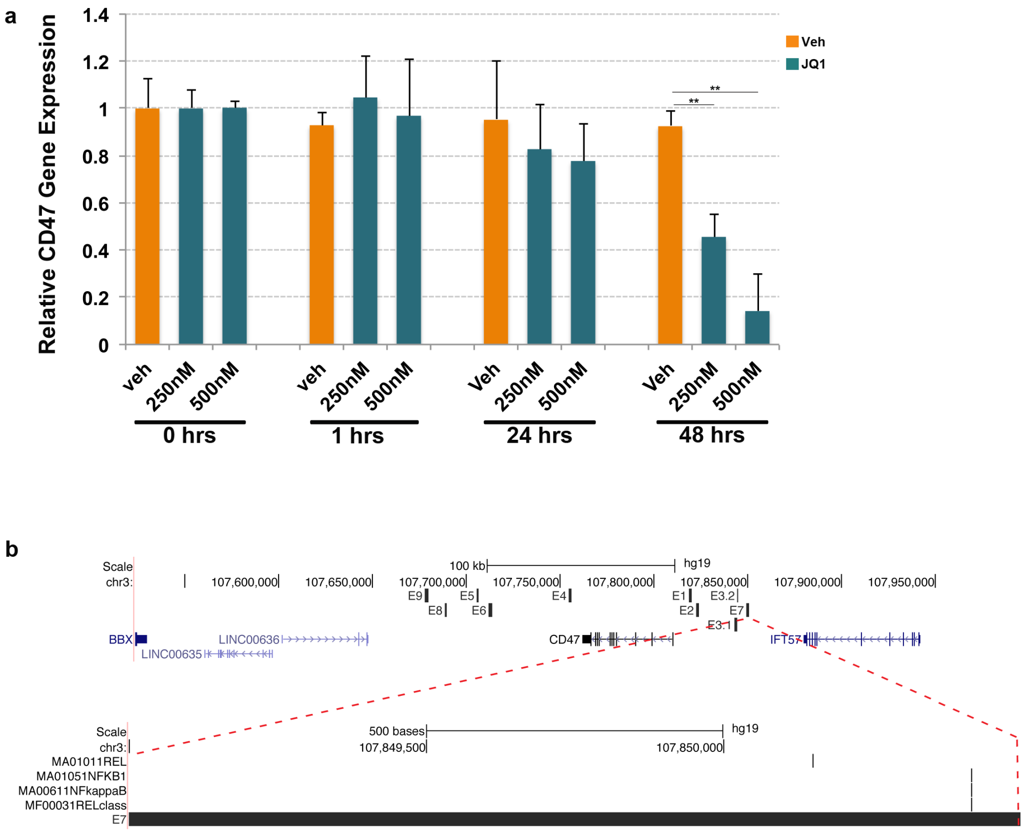

Supplementary Figure 9. A functional upstream *CD47* SE in Jurkat (T-ALL) has predicted binding sites for NFKB family members within the E7 constituent (a) Treating Jurkat cells for 48 hours with the BRD4 inhibitor JQ1 at different concentrations reduces *CD47* expression. N=3 samples. Values represent mean + s.d. Student's unpaired t test for independent samples was performed. **P <0.01. (b) Schematic showing the location of E7 active enhancer within the *CD47* genomic locus, and the location of binding motifs within E7 predicted by PIQ. NFKB1 and other factors belonging to the NFKB family are predicted to bind to E7.

Supplementary Table 1. Datasets used for the identification of super-enhancers

Cell type	H3K27ac	Input
HMEC	GSM733660	GSM733668
BT 549	GSM1589476	GSM1589475
MDA-MB-468	GSM1589470	GSM1589471
MDA-MB-231	GSM1204474	GSM1204476
T47D	GSM1589474	GSM1589473
ZR-75-1	GSM1589472	GSM1589477
HCC1954	GSM721136	GSM721139
MCF7	GSM946850	GSM945859
CD3+ T cell	GSM1058764	GSM1058789
Jurkat	GSM1296384	GSM1296386
MOLT3	GSM1519644	GSM1519645
Sigmoid Colon	GSM915331,GSM910559	GSM906422
HCT-116	SRR504923	GSM749774
Pancreas	GSM1013129,GSM906397	GSM1013172,GSM90641[illegible]
Panc1	SRR353689	GSM818828
DND41	GSM1003462	GSM1003558
GLC16	GSM1526704	GSM1526701
H2171	GSM894067	GSM894092
H69	GSM1526703	GSM1526705
H82	GSM1526706	GSM1526702
HeLa	GSM733684	GSM733659
HepG2	GSM733743	GSM733732
K562	GSM733656	GSM733780
Kelly	GSM1532401	GSM1532403
LnCAP	GSM686937	SRR122337
MM1S	GSM894083	GSM894087
SHSY5Y	GSM1532408	GSM1532410
VACO 400	GSM883684	GSM883666
VACO 503	GSM883682	GSM883671
VACO 9M	GSM883683	GSM883674
NGP	GSM2037793	Not submitted yet
P265 Primary DLBCL	GSM1254210	GSM1254211
P286 Primary DLBCL	GSM1254212	GSM1254213
P397 Primary DLBCL	GSM1254202	GSM1254215
P448 Primary DLBCL	GSM1254216	GSM1254217
DHL6	GSM1254194	GSM1254195
HBL1	GSM1254196	GSM1254197
LY3	GSM1254198	GSM1254199
LY4	GSM1254200	GSM1254201
TOLEDO	GSM1254202	GSM1254203
P493	GSM1036405	GSM1036408

Colo741	GSM1296640	GSM1296639
A673	GSM1517563	GSM1517568
U2OS	GSM1356567	GSM1231607
LS174T	GSM1365900	GSM1197323
HT29	GSM1296643	GSM1296642
RPMI-8402	GSM1442003	GSM957613

Supplementary Table 2. Primers used for the amplification of distal putative CD47 enhancers

	Forward (5'-3')	Reverse (5'-3')
E1-CD47	TAGTGTGCCAGAAACAGC	TCTAATACTGATCTCATTGC
E2-CD47	AATTACAACATATCTCTCTGC	ACTGTTGAAATGGGGAAAACC
E3.1-CD47	CTGTATTTCCATTCTCATGC	TACTTTTGTCATCAGCTTCC
E3.2-CD47	ACTCACTTACATGCATGC	ACACTATCTCCAAATAAGG
E4-CD47	AAAGGGAACCTAAACTGTCC	TACTTTGATTTGTAGACCATCG
E5-CD47	TCAGACTTAGTTTGTAGATGG	ATAACACAGGGAATAGAAGC
E6-CD47	AATACATTCAAGTGGTCAGG	TCCTTCTCTGTGCAAACC
E7-CD47	ACAGACTTCCAGCAGAGAC	TTCCATGTACATTTCATGG
E8-CD47	TTTCCTACTTTCATTCATCCTA	CTAATTCAGTCAGTCCTGATG
E9-CD47	CAGCATTACCTCTACCATTCT	TTAATCTATAAATATCAATTTGCTG
